# Bacterial Epigenomics: Coming of Age

**DOI:** 10.1128/mSystems.00747-21

**Published:** 2021-08-17

**Authors:** Pedro H. Oliveira

**Affiliations:** a Génomique Métabolique, Genoscope, Institut François Jacob, CEA, CNRS, Université Évry, Université Paris-Saclay, Évry, France

**Keywords:** methylation, metaepigenomics, holoepigenomics, antimicrobial, single cell, epigenetics

## Abstract

Epigenetic DNA methylation in bacteria has been traditionally studied in the context of antiparasitic defense and as part of the innate immune discrimination between self and nonself DNA. However, sequencing advances that allow genome-wide analysis of DNA methylation at the single-base resolution are nowadays expanding and have propelled a modern epigenomic revolution in our understanding of the extent, evolution, and physiological relevance of methylation. Indeed, as the number of mapped bacterial methylomes recently surpassed 4,000, increasing evidence supports roles for methylation in gene expression regulation, virulence, and host colonization, among others. In this paper, I summarize lessons taken from high-dimensional methylome data analyses and recent efforts that we and others are developing to leverage such findings into meaningful biological insights and overarching frameworks. Ultimately, I highlight anticipated research avenues and technological developments likely to unfold in the coming years.

## COMMENTARY

Epigenomics refers to the systematic analysis of heritable, yet reversible, molecular modifications to both DNA and chromatin, the most extensively studied of which is DNA methylation. In bacteria, three major forms of DNA methylation have been detected: N6-methyladenine (6mA, the most abundant), N4-methylcytosine (4mC), and 5-methylcytosine (5mC) (1). These marks are mediated by DNA methyltransferases (MTases) associated with restriction-modification (R-M) systems or by orphan MTases (lacking a cognate restriction endonuclease [REase]) (2, 3). Propelled by recent progresses in third-generation sequencing technologies—single-molecule real-time sequencing (SMRT-seq) by Pacific Biosciences and nanopore sequencing by Oxford Nanopore Technologies—more than 4,000 methylomes have been mapped to date (4, 5). As a consequence, the field of bacterial epigenomics is witnessing a remarkable expansion beyond single methylome analyses to the realm of multi-omic data integration. As an example, we recently performed a large-scale DNA methylome and transcriptome analysis in the key human pathogen Clostridioides difficile and found a conserved orphan MTase whose inactivation impacted fundamental phenotypes involved in its transmission to a host (6). Such findings add to the growing number of studies integrating multi-omics profiling to identify putative epigenetic regulation networks. Fueled by this exciting momentum, my laboratory combines high-throughput (epi)genomic technologies and bioinformatic approaches to address outstanding questions put forward in the bacterial epigenomics field. What phenotypes are impacted by DNA MTases? Can we develop novel antimicrobial strategies by harnessing methylation systems? What is the interplay between stress adaptation and the stable inheritance of certain DNA methylation marks? In this paper, I describe my vision on how these lines of study will unfold and call out the challenges ahead.

## THE UNDERRATED BACTERIAL 5mC METHYLOME

Bisulfite sequencing has traditionally been regarded as the gold standard approach enabling genome-wide 5mC mapping. This particularly holds true for eukaryotes, where 5mC is the most common DNA modification and is associated with a variety of biological phenomena such as gene silencing, genomic imprinting, X chromosome inactivation, RNA splicing, and silencing of transposable elements (7). Perhaps due to the lower genomic predominance and anticipated minor role of 5mC in bacterial gene regulation, the use of bisulfite sequencing has been rather limited in bacteria (8–10). Moreover, 5mC detection by third-generation sequencing technologies has been hampered either by the need for very high sequence coverage/ten-eleven translocation dioxygenase hypermodification in SMRT-seq (11) or by the limited availability of methods to perform *de novo* fine mapping of methylation type and recognition motif for nanopore sequencing (12). Notwithstanding, 5mC methylation is emerging as an important mechanism in bacterial epigenetics, as recent studies have thrown light on previously underappreciated roles in virulence and host adaptation. Some notorious examples include the control of bacterial cell shape, adherence to host cells, natural competence for DNA uptake, and envelope formation (9, 13). Perhaps more surprising was the recent finding of a substantial number of highly conserved 5mC bacterial MTases for which little is known regarding the underlying epigenetic mechanisms regulating cellular phenotypes (14). Another outstanding question concerns the origin and evolution of DNA methylation across the tree of life. In the case of 5mC, despite the different target sequence contexts in which it takes place, methylation is established and maintained by a family of DNA MTases that share a catalytic domain containing 10 conserved small motifs, suggesting a common origin (15). Also, the impact of 5mC on the intrinsic structure and mechanical properties of DNA (reduced flexibility and widening of the major groove) is expected to be consistent across different organisms, which may nevertheless take advantage of such conformational changes under very distinct genetic conditions. The extent to which such DNA structural changes are a function of sequence context and how they impact recognition by DNA-binding proteins are still unclear. Hence, understanding the full significance of 5mC methylation, its functional consequences, and evolution remains an exciting challenge for the future.

## HARNESSING METHYLATION SYSTEMS AS AN ANTIMICROBIAL STRATEGY

Orthodox Type IIP R-M systems are composed of one homodimeric or homotetrameric REase and one monomeric MTase and are able to operate separately and independently from each other. Such a feature allows these systems to behave as toxin-antitoxin addiction modules and facilitate programmed cell death by postsegregational killing (16). Given these observations, it is reasonable to ask whether bacterial R-M systems could be exploited for clinical purposes. In particular, could we envisage an antimicrobial chemotherapeutic strategy based on molecules that selectively interfere with the R-M balance through binding to the MTase or by enhancing its rate of proteolysis? Such a strategy would result in the loss of protection provided by epigenetic methylation, followed by cleavage of chromosomal DNA by the cognate REase and ultimately cell death. One downside of this approach is that R-M genes are frequently exchanged between bacteria by horizontal gene transfer and evolve very quickly, making it more likely to be used as a narrow-spectrum therapy against a particular species or emergent strain where the targeted R-M system would be significantly conserved and expressed. An alternative strategy would be to target orphan MTases that are conserved at a given taxonomic rank (e.g., species level). The former are typically encoded by core/quasicore genes and frequently found to be conditionally essential. One interesting possibility for MTase inhibition would be to use analogs of the methyl donor *S*-adenosyl-l-methionine (SAM) or bisubstrate inhibitors that simultaneously target SAM- and substrate-binding sites (17). Such a scenario was recently proposed for the core MTase CamA of C. difficile (18). Another example is that of the well-characterized Escherichia coli Dam enzyme, whose inhibition reportedly weakens bacterial pathogenicity *in vivo* (19, 20). Dam methylation was also found to play a role in drug potentiation, by curbing the therapeutic activity of the β-lactam and quinolone classes of antibiotics (21). In this view, Dam represents an attractive target for epigenetic inhibition of the multiple biological processes that it regulates (e.g., virulence), as it lacks mammalian homologs while being conserved in several enteric pathogens. While some selective inhibitors of Dam were previously proposed (22), there have been no further advances over the past decade. Since *camA* and *dam* are part of a much larger list of 145 genes recently reported in a study investigating highly conserved MTases in bacteria (14), I foresee a renewed interest in the exploitation of such targets for the development of next-generation epigenetic drugs (23–25).

## META- AND HOLOEPIGENOMICS

**FIG 1:**
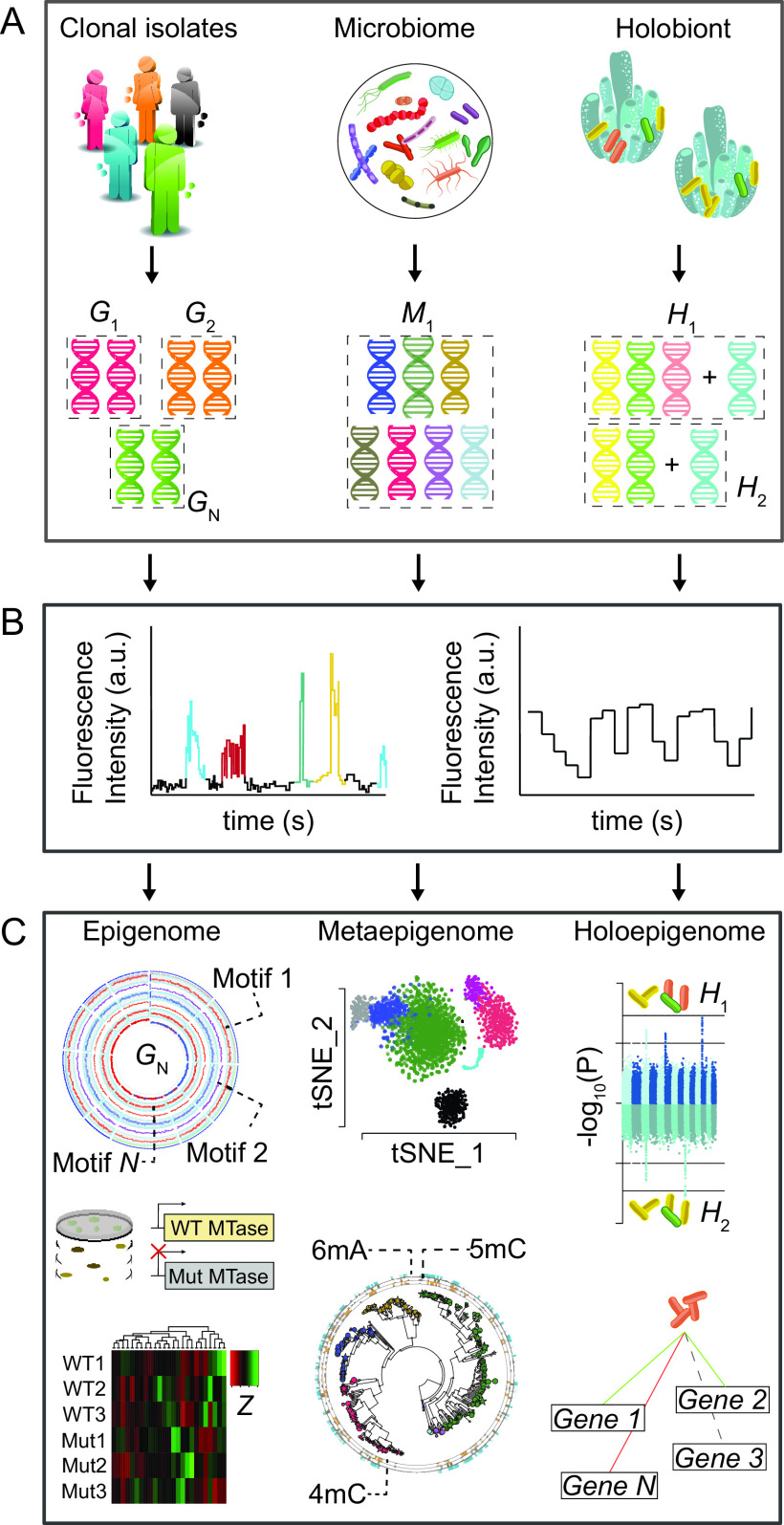
Approaches to study bacterial methylomes from clonal isolates, microbiomes, and holobionts. (A) Although a large abundance of methylomes profiled to date belongs to genome (*G*) isolates, there is a growing interest in the analysis of microbiome (*M*) and holobiont (*H*) methylomes. (B) Recent progress in third-generation sequencing technologies (e.g., SMRT-seq and nanopore sequencing) has enabled direct genome-wide detection of methylated positions and target motifs. (C) Relevant functional information on the epigenome can be obtained by targeted mutagenesis of DNA MTases. A comprehensive global transcriptome and functional profiling by RNA-seq offers the opportunity to further dissect the range of differentially expressed genes in a methylation-free strain. For metaepigenomes, nonlinear dimensionality reduction algorithms such as the t-distributed stochastic neighbor embedding (t-SNE) are a possible option to visualize and interpret methylation features across multiple metagenomic contigs. A phylogenetic representation of methylation systems’ density across several metagenome-assembled genomes may also provide clues into the interplay between DNA methylation and factors unique to the environment of each community. In holoepigenomes, genome-wide analysis of CpG site methylation differences between multiple hosts (as shown in the Manhattan plots of *P* values) may provide insight into the network of host genes whose expression is being significantly modulated by the presence of certain symbionts.

It is estimated that more than 99% of the potentially 10^11^ to 10^12^ species that make up all microbial diversity on Earth remain unexplored to date and that only a small fraction can be culturable under standard laboratory conditions (26). Culture-independent techniques such as metagenomic sequencing have provided a greater depth of understanding of the biodiversity and functional capabilities of microbial communities. The introduction of third-generation sequencing technologies has substantially improved metagenome assemblies and holds the potential to change our understanding of the hidden diversity of methylomes across different ecological niches (Fig. 1). In two recent metaepigenomic studies performed in aquatic ecosystems, important advances were made both in the finding of previously undescribed target methylation sites and in the understanding of the coevolutionary history of methylation systems and host genome (27, 28). These findings add to recent bioinformatic developments exploring endogenous epigenetic barcodes as complements to coverage and composition features in order to improve strain-level resolution of metagenomes and link mobile genetic elements to their host genomes in microbial samples (29). It is expected that in the next few years, metaepigenomic analyses of bacteria from different ecological niches will significantly deepen our understanding on the evolution of methylation systems and on the impacts of DNA methylation in shaping the composition of such niches. More broadly, the systematic search for antiphage defense hot spots in metagenomic data sets is expected to uncover novel immune systems, with the potential to be adapted into useful molecular tools. While the metaepigenome encompasses the ensemble of epigenetic changes in a community within a nonliving environment, there is an increasing interest in studying the holoepigenome, which by definition implies an epigenetic interaction between the host and its symbionts (the holobiont) (Fig. 1). Such interactions can affect key biological processes of both hosts and microorganisms and have the power to shape their coevolution. For example, dysbiosis and reduction of microbial diversity can change the proportion of metabolites acting as regulators of DNA and histone modifications in the host. Alternatively, the secretion or injection/translocation of nucleus-targeted effectors—termed nucleomodulins—from a bacterial pathogen into the host cytosol can subvert the host epigenome through interference with histone and DNA modifications, regulation of transcription, interference on the cell cycle, and regulation of cell signaling pathways. For example, the nucleomodulins Mhy from Mycoplasma hyorhinis and Rv2966c from Mycobacterium tuberculosis are capable of acting as mammalian DNA MTases and regulate proliferation-specific pathways (30). Hence, it is foreseeable that the next years will bring additional research on nucleomodulin diversity in bacterial pathogens and a better understanding of the mechanisms used for nuclear trafficking and modulation of the host genome.

## VIEW FROM A SINGLE-CELL PERSPECTIVE

Exposure of clonal bacterial populations to environmental changes, stress, and other stimuli results in methylome alterations that modulate global gene expression patterns. Such nongenetic diversification can in turn lead to the emergence of phenotypically heterogeneous subpopulations, in which some persister cells have a better ability to withstand the change. In recent years, important technical advancements in single-cell isolation, whole-genome/transcriptome amplification, and high-throughput sequencing are paving the way for resolving cell-to-cell multi-omic heterogeneity at unprecedented resolution. However, the development of efficient high-throughput single-cell solutions for microbial systems has lagged behind those for eukaryotes (31), mainly due to their low DNA/mRNA content, difficult lysis/permeabilization of cell walls and membranes, and lack of polyadenylation of bacterial mRNA, which limits its separation from rRNA. Since long-read sequencing technologies require a relatively large amount of starting genomic DNA for library preparation, it is conceptually challenging to perform single-cell analysis. Moreover, such technologies rely on a consensus sequence obtained from a cell population and lack the resolution required to perceive epigenetic heterogeneity. In this sense, a recently proposed bioinformatics tool allows performing single-molecule characterization of epigenetic heterogeneity in bacterial methylomes using SMRT-seq (32). One interesting research avenue that would greatly benefit from an in-depth bacterial methylome tracking at the single-cell level is the one dealing with genetic assimilation. The latter essentially assumes that a stress-induced nongenetic change in phenotype can, during the course of selection and over multiple generations, become genetically encoded. This necessarily raises a few outstanding questions: (i) is this genetic assimilation aimed at maintaining stress-related epigenetic landscapes? (ii) are the observable changes in gene expression directly modulated by the acquisition of a particular subset of DNA methylation marks? Although recent studies have begun to provide insight into this topic (33), we will need to wait for further advances in long-read technologies applied to single-cell sequencing, in order to identify the missing pieces of what appears to be not only a complex puzzle of epigenetic-mediated persistence but also a promising gateway for the development of novel antibacterial drugs.
