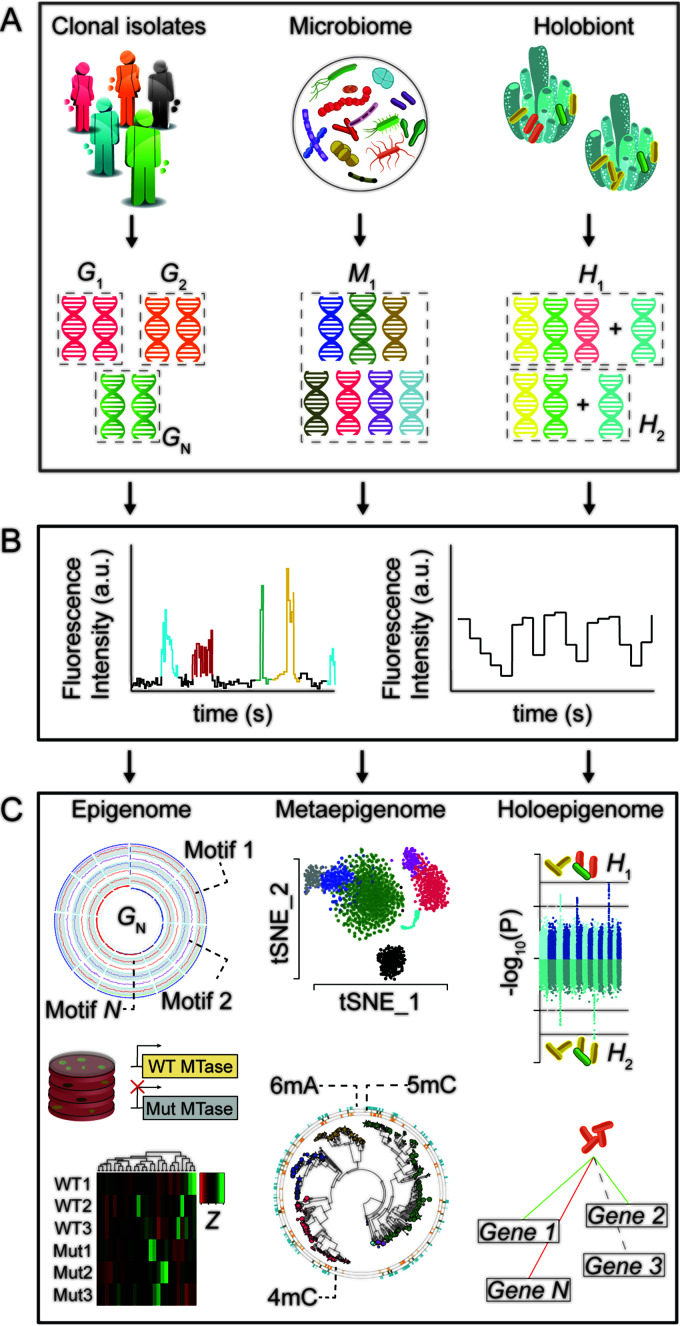# Correction for Oliveira, “Bacterial Epigenomics: Coming of Age”

**DOI:** 10.1128/mSystems.01075-21

**Published:** 2021-09-07

**Authors:** Pedro H. Oliveira

**Affiliations:** a Génomique Métabolique, Genoscope, Institut François Jacob, CEA, CNRS, Université Évry, Université Paris-Saclay, Évry, France

## AUTHOR CORRECTION

Volume 6, no. 4, e00747-21, 2021, https://doi.org/10.1128/mSystems.00747-21. Page 4: Fig. 1 should appear as shown below.

**Figure 1:**